# Acute Localized Exanthematous Pustulosis in a 5-Year-Old Boy With Zosteriform Distrubiton

**DOI:** 10.7759/cureus.65402

**Published:** 2024-07-26

**Authors:** Kerem Balan, Furkan Aydın, Neslihan Akdogan

**Affiliations:** 1 Dermatology, Hacettepe University, Ankara, TUR

**Keywords:** pediatrics, allergy, zosteriform, drug eruptions, acute localized exanthematous pustulosis

## Abstract

Acute localized exanthematous pustulosis (ALEP) is typically a benign drug reaction that occurs shortly after drug intake. We report a five-year-old male who developed a localized, zosteriform pustular rash on his back 10 days after treatment with oseltamivir and ceftriaxone for an upper respiratory tract infection. The lesions, which appeared three days prior to presentation, were mildly itchy and resolved completely without scarring within two days of treatment with topical betamethasone valerate and fusidic acid. No bacterial growth was detected in the pustular culture. This case highlights the rare occurrence of ALEP in a pediatric patient and suggests variations from the EuroSCAR diagnostic criteria, which usually indicate a 72-hour onset post-drug intake, noting instead a 7-14 day onset in atypical cases.To best of our knowledge, This is the first report of ALEP presenting with a zosteriform distribution.

## Introduction

Acute localized exanthematous pustulosis (ALEP) represents a confined variant of acute generalized exanthematous pustulosis. It manifests with a sudden onset of numerous small, sterile pustules that are non-follicular and pinhead-sized, typically occurring after the administration of a drug [1]. Although antibiotics and anti-inflammatories are the most commonly implicated agents in etiology, cases have been reported even with insect bites as triggers [1,2]. It is a rare but benign condition characterized by its rapid onset and resolution [1]. ALEP is more commonly observed in young adults but it is an exceptionally rare condition in the pediatric age group [3]. We presented a five-year-old patient who developed localized pustular rash following oseltamivir and ceftriaxone treatment for upper respiratory tract infection.

## Case presentation

**Figure 1:**
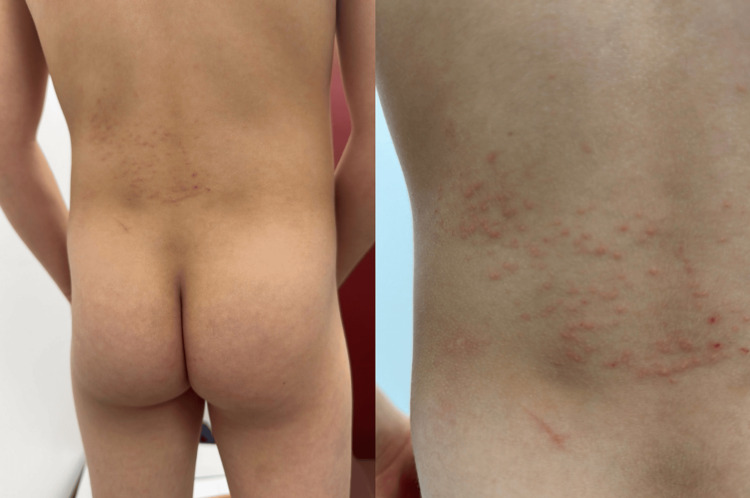
Erythematous based pustules in a dermatomal distribution on the lower back.

**Figure 2:**
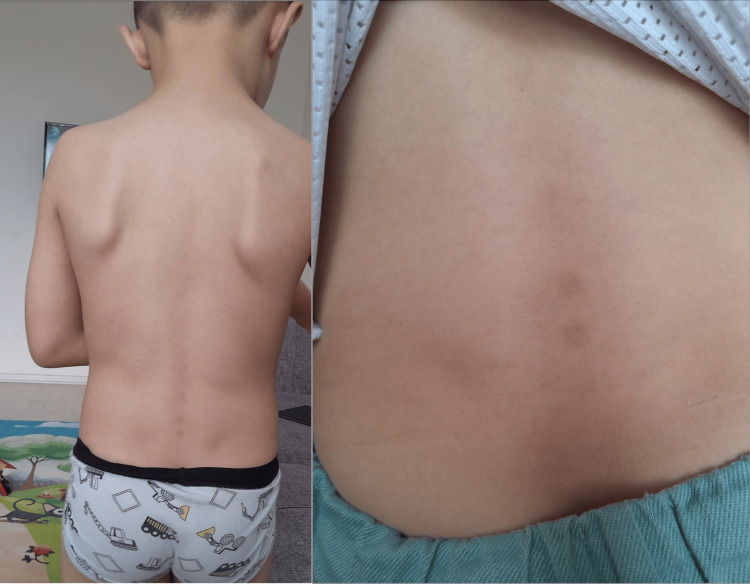
After one week of topical treatment with betamethasone valerate (1 mg/g) and fusidic acid (20 mg/g)

A five-year-old male presented to us with localized, zosteriform distributed pustular rash on his back. Approximately 20 days ago, the patient was initiated on oseltamivir due to an upper respiratory tract infection. As the patient's symptoms persisted for 10 days without improvement, ceftriaxone therapy was added. The ceftriaxone treatment was completed over 10 days and then discontinued. The patient began experiencing a rash around the seventh day of ceftriaxone treatment. The patient presented to our clinic on the approximately third day after the onset of the rash and had mild itching but no pain. Upon evaluation, localized non-follicular pustules on an erythematous base, occasionally clustered, were observed on the patient's back (Figure 1). There was no history of any contact exposure in this area. The absence of lesions in other parts of the patient's body was noted. Due to the localized nature of the lesion, ALEP, miliaria, and infectious pustules were primarily considered. However, the preliminary diagnosis of miliaria was ruled out because the patient had no history of heat exposure, fever, sweating, or rash in other areas, and patient presented during mid-winter. Cultures were taken from the pustular discharge. In addition to the topical betamethasone valerate (1 mg/g) treatment, a combination preparation of topical fusidic acid (20 mg/g) was prescribed due to the inability to exclude a superficial skin infection. The patient was scheduled for a follow-up to evaluate the culture results and treatment response. At the one-week evaluation, the lesions were found to have completely regressed (Figure 2). According to information obtained from the family, the lesions had completely disappeared by the second day of topical treatment. Culture of the pustul was reported as sterile. Upon observing the complete regression of the patient's lesions, the topical treatment was discontinued after one week of therapy. 

## Discussion

While ALEP is more common in young adult females, it is more prevalent in males in the pediatric age group [3]. Our case involved a pediatric male patient. Although the literature previously indicated that ALEP typically begins 72 hours after drug intake based on revised EuroSCAR diagnostic criteria for ALEP, it has been noted that in atypical cases, ALEP can start 7-14 days after drug intake [4]. In our patient, due to the rapid onset and resolution of lesions, along with no growth in the culture, the erythematous base of the lesions, and clustering of pustules, the diagnosis of ALEP was made. The development of lesions approximately one week after drug intake demonstrates a variation from revised EuroSCAR criteria for ALEP. Although discontinuing the offending medication and moisturisers are often sufficient for treatment, topical or oral steroids have been found to be particularly beneficial for reducing pruritus [1].

The following are the revised EuroSCAR criteria for ALEP [4].

1. Localised numerous small (1-3 mm) clustered non-follicular pustules

2. Background erythema

3. Negative microbiology

4. Acute onset (<72 h) after medication

5. Resolution (with post-pustular desquamation) within 14 days of discontinuing medication

## Conclusions

As research on ALEP expands, it becomes increasingly important to refine both diagnostic criteria and treatment protocols to address the variability seen in clinical presentations. Our case provides significant insights that contribute to the standardization of ALEP management, especially in younger patients presenting with atypical features. Future investigations should focus on exploring the varied manifestations of ALEP and validating these observations through larger studies to improve diagnostic precision and therapeutic outcomes.
